# Risk of cancer in patients with heart failure who use digoxin: a 10-year follow-up study and cell-based verification

**DOI:** 10.18632/oncotarget.17410

**Published:** 2017-04-25

**Authors:** Min-Huey Chung, Yi-Wen Wang, Yung-Lung Chang, Shih-Ming Huang, Wei-Shiang Lin

**Affiliations:** ^1^ Graduate Institute of Nursing, College of Nursing, Taipei Medical University, Taipei 110, Taiwan, Republic of China; ^2^ Department of Biology and Anatomy, National Defense Medical Center, Taipei City 114, Taiwan, Republic of China; ^3^ Department of Biochemistry, National Defense Medical Center, Taipei City 114, Taiwan, Republic of China; ^4^ Department of Medicine, Division of Cardiology, Tri-Service General Hospital, National Defense Medical Center, Taipei City 114, Taiwan, Republic of China

**Keywords:** heart failure, digoxin, cancer, cell-based strategy, personalized medicine

## Abstract

Heart failure (HF) is the leading cause of death in the world and digoxin remains one of the oldest therapies for HF. However, its safety and efficacy have been controversial since its initial use and there is uncertainty about its long-term efficacy and safety. Recently, the repositioning of cardiac glycosides is to function in anti-tumor activity via multiple working pathways. It is interesting to compare the potential effects of digoxin in clinical patients and cell lines. First, we analyze patient information retrieved from the National Health Insurance Research database of Taiwan between January 1, 2000 and December 31, 2000. This retrospective study included a study cohort (1,219 patients) and a comparison cohort. Our analytical data suggested that patients taking digoxin are at an increased risk of cancers, including breast, liver, and lung cancers, during the 10-year follow-up period. In contrast to the anti-tumor function of digoxin, we further examined the potential pathway of digoxin via the cell-based strategy using several breast cancer cell lines, including MCF-7, BT-474, MAD-MB-231, and ZR-75-1. Digoxin consistently exerted its cytotoxicity to these four cell lines with various range of concentration. However, the proliferation of ZR-75-1 cells was the only cell lines induced by digoxin and the others were dramatically suppressed by digoxin. The responsiveness of SRSF3 to digoxin might be involved with cell-type differences. In summary, we combined a cohort study for digoxin treatment for HF patients with a cell-based strategy that addresses the translation issue, which revealed the complexity of personalized medicine.

## INTRODUCTION

Heart failure (HF), characterized by pulmonary congestion, dyspnea, and fatigue, is a clinical syndrome in which the myocardial pump function is inadequate to maintain and support the physiological requirements of an individual. Digoxin, as well as digitoxin, is an ancient and effective therapy for congestive HF via the inhibition of the plasma membrane Na^+^/K^+^-ATPase [1, 2]. The signaling characteristics of Na^+^/K^+^-ATPase are distinct from its ion pumping activity and activate associated downstream signaling pathways [1, 3]. In addition to the sustained increase in the level of intracellular calcium for immunogenic cell death and apoptosis of tumor cells [4–7], digoxin also exerts its anti-tumor functions via multiple mechanisms, including at the post-transcriptional and translational stages [8]. However, there is uncertainty about its long-term efficacy and safety for the treatment of HF.

The role of big data in healthcare is to build better health profiles and better predictive models around patients so that doctors can predict epidemics, cure disease, improve the quality of life, and avoid preventable deaths [9]. There are many retrospective studies, including epidemiological and clinical, that have investigated whether the use of digoxin would increase or decrease incidences of several tumors [10–18], such as breast and prostate cancers (Table 1). In most cases, the use of digoxin decreased their incidence. Breast cancer was the only increased case, suggesting that digoxin resembles estrogen chemically and may have an estrogenic effect [12, 15–17]. Furthermore, the work of Biggar's laboratory has shown that consistently increased risks from digoxin use is marginally higher for estrogen receptor (ER) positive breast cancers [12, 15, 16].

**Table 1:** Retrospective clinical studies assessing the impact of digoxin in breast and prostate cancers

Cancer Type	Number of patients	HR, OR or RR, (95% CI), p value	Notes	Ref
	175	Not available	Use decreased death rate	10
	324	1.30, (1.14–1.46)	Use increased risk among postmenopausal women	11
Breast	104,648	1.39, (1.32–1.46)ER^+^: 1.35, (1.26–1.45)	Use increased risk, mainly of developing ER^+^ lesions	12
	1,219	1.30, (1.05–1.62) p < 0.001	This study	
	1,006	p = 0.046	Inverse correlation between use and survival	13
Prostate	47,884	Regular user: 0.54, (0.37–0.79) p < 0.001Users for > 10 y: 0.76, (0.61–0.95)	Use (in particular ≥ 10 y) decreased risk	14
	1,219	0.75, (0.41–1.37)	This study	

We take advantage of related clinical information regarding claims from the National Health Insurance (NHI) program, which was initiated by the Taiwanese government in 1995, is a compulsory single-payer system, and covers over 98% of the 23 million inhabitants in Taiwan [19]. Our retrospective study includes a study cohort and a comparison cohort. Our analytical data shows that the increased risk of cancers by digoxin during a 10-year follow-up period was found in breast, liver, and lung cancers. Finally, we examined the potential role of digoxin in the proliferation of breast cancer cell lines. The combination of this information and cell-based strategy could allow us to understand the working mechanisms as well as the side effects of clinical drugs.

## RESULTS

### Digoxin and subsequent risk of cancers

In 2000, we identified 1,219 pHF-Digo, patients with heart failure who have taken digoxin, and 2,942 pHF, the patients with heart failure who had not taken digoxin, in the comparison group (Table 2). Over half of pHF-Digo were older than 65 years-old, 55.11% were female patients and 84.76% had a monthly income < US$ 640 (< NT$ 20,000). After analyzing the characteristics of digoxin exposure in patients with heart failure in Taiwan in 2000, the variables of patients’ age, income, region, and Charlson Comorbidity Index (CCI) were significant. Table 3 shows the incidence of patients with cancers during the 10-year follow-up period for pHF-Digo. The incidence of cancers among pHF-Digo was higher than in pHF (11.66 vs. 10.44 per 100 person-years). pHF-Digo had a significantly higher incidence of being diagnosed with cancers compared to pHF for each of the previously named variables. In addition, the AHR of males was significant, patients ≥ 65 years-old, < 45 years-old; and the group with a CCI score ≥ 3 are significant.

**Table 2:** Characteristics of Digoxin exposure in heart failure patients in Taiwan from 2000/01/01-/12/31 (N=4,161)

Variable	All Heart Failure		Heart Failure				p-value
	(n=4,161)		Digoxin (n=1,219)		Non-Digoxin (n=2,942)		
	n	%	n	%	n	%	
**Age (years)**							<0.001
<45	398	9.57	71	5.83	327	11.12	
45–64	1,428	34.32	340	27.89	1,088	36.98	
≥65	2,335	56.11	808	66.28	1,527	51.90	
**Gender**							0.104
Female	2,293	55.11	648	53.16	1,645	55.91	
Male	1,868	44.89	571	46.84	1,297	44.09	
**Incomes (US$)**							<0.001
≥1281 (≥ NT$ 40,000)	204	4.90	46	3.77	158	5.37	
640–1280 (NT$ 20,000–39,999)	430	10.34	93	7.63	337	11.46	
<640 (< NT$ 20,000)	3,527	84.76	1,080	88.60	2,447	83.17	
**Region**							<0.001
Northern	1,676	40.28	453	37.16	1,223	41.57	
Center	1,191	28.62	399	32.73	792	26.92	
South	1,141	27.42	317	26.00	824	28.01	
East	153	3.68	50	4.20	103	3.50	
**Urbanization**							0.124
Urban	2,118	50.90	596	48.89	1,522	51.73	
Suburban	1,546	37.16	461	37.82	1,085	36.88	
Rural	497	11.94	162	13.29	335	11.39	
**CCI**							<0.001
<3	1,357	32.61	328	26.91	1,029	34.98	
≥3	2,804	67.39	891	73.09	1,913	65.02	
**Total**	4,161	100.00	1,219	100.00	2,942	100.00	

CCI, Charlson Comorbidity Index scores.

**Table 3:** Cancer risk associated with digoxin use in heart failure patients in Taiwan

Variable	HF Patient with Digoxin			HF Patient with Non-Digoxin			Hazard ratio and 95% CI	
	Cases	PY	Incidence	Cases	PY	Incidence	CHR^a^	AHR^b^
**Age (years)**								
<45	5	689.4	0.73	26	3408.8	0.76	Ref.	Ref.
45–64	46	3170	1.45	147	10973.8	1.34	1.83(1.25–2.67)*	1.69(1.16–2.48)*
≥65	148	6593.9	2.24	307	13790	2.23	3.13(2.17–4.50)**	2.61(1.80–3.80)**
**Gender**								
Female	110	5585.6	1.97	230	16078	1.43	Ref.	Ref.
Male	89	4867.7	1.83	250	12094.6	2.07	1.29(1.11–1.50)**	1.27(1.09–1.48)*
**Incomes (US$)**								
≥1281(≥ NT$ 40,000)	6	443.6	1.35	23	1582.8	1.45	Ref.	Ref.
640–1280 (NT$ 20,000–39,999)	11	865.4	1.27	46	3380.9	1.36	0.94(0.60–1.47)	0.87(0.56–1.37)
<640 (< NT$ 20,000)	182	9144.3	1.99	411	23208.9	1.77	1.32(0.91–1.91)	1.00(0.68–1.48)
**Region**								
Northern	77	3831	2.01	211	11659.6	1.81	Ref.	Ref.
Center	65	3432.6	1.89	115	7718.5	1.49	0.86(0.72–1.04)	0.81(0.67–0.99)*
South	49	2752.5	1.78	143	7803.4	1.83	0.98(0.82–1.17)	0.91(0.75–1.10)
East	8	437.2	1.83	11	991.1	1.11	0.71(0.45–1.14)	0.71(0.45–1.14)
**Urbanization**								
Urban	100	5109	1.96	243	14668.1	1.66	Ref.	Ref.
Suburban	71	3957	1.79	179	10320	1.73	1.01(0.86–1.19)	0.96(0.81–1.14)
Rural	28	1387.3	2.02	58	3184.5	1.82	1.09(0.86–1.38)	1.08(0.85–1.37)
**CCI**								
<3	36	3063	1.18	105	10470.7	1.00	Ref.	Ref.
≥3	163	7390.3	2.21	375	17701.9	2.12	2.13(1.77–2.57)**	1.86(1.54–2.25)**
**All**	199	10453.3	1.90	480	28172.6	1.70	1.16(0.98–1.37)	1.04(0.88–1.23)

PY, person-years at risk per 100 person-years, HF, Heart Failure

a. CHR: Crude hazard ratio was calculated by using stratified Cox proportional hazards regression

b. AHR (adjusted hazard ratio): Controlling the variables of age, gender, income, region, urbanization, cci, *: p<0.05 **: p<0.001

### Cancers and subsequent risk of digoxin

During 2000/1/1–12/31, to further analyze the relation between cumulative defined daily dose (DDD) of digoxin per year and heart failure patients with cancers, we identified 1,219 heart failure patients with digoxin dosage from all heart failure patients and divided them into 2 groups by the median of cumulative per year dose: 140 (Table 4). After adjusting for age, gender, income, region, urbanization, and CCI, the results revealed that no significant differences were found in the Cox proportional hazard regression model in each group. Table 5 shows the incidence of cancers during the 10-year follow-up period for pHF-Digo. We found that the adjusted hazard ratio (AHR) for breast cancer among pHF-Digo was particularly high (AHR = 15.28, 95% CI -11.72–42.29) compared with the control group. After adjusting for age, gender, income, region, urbanization, and CCI, the results revealed significant differences were found for breast cancer (AHR = 1.30, 95% CI 1.05–1.62) and liver cancer (AHR = 1.29; 95% CI 1.03–1.62). There was also an increased risk of lung cancer among study patients compared with the control group (CHR (crude hazard ratio) = 1.38; 95% CI 1.11–1.7). However, there was no significantly increased risk of colorectal and gynecological cancers or no significantly decreased risk of prostate, bladder, and kidney cancers.

**Table 4:** Relation between cumulative defined daily dose of digoxin per year and HF patients with cancers

Dose	No. of Cases	PY	Incidence^a^	CHR (95% C.I.)	AHR (95% C.I.)
**ALL**					
DDD<140	93	5058.4	1.84	Ref.	Ref.
DDD≥140	106	5394.9	1.96	1.06 (0.80–1.40)	1.12 (0.85–1.49)
**Age<45**					
DDD<140	3	292.9	1.02	Ref.	Ref.
DDD≥140	2	396.5	0.50	0.51 (0.08–3.02)	0.21 (0.02–2.87)
**45≤Age<65**					
DDD<140	23	1571	1.46	Ref.	Ref.
DDD≥140	23	1599	1.44	0.98 (0.55–1.74)	1.05 (0.59–1.89)
**Age ≥65**					
DDD<140	67	3194.5	2.10	Ref.	Ref.
DDD≥140	81	3399.4	2.38	1.12 (0.81–1.55)	1.17 (0.84–1.63)
**Female**					
DDD<140	47	2679.9	1.75	Ref.	Ref.
DDD≥140	63	2905.7	2.17	1.24 (0.85–1.81)	1.32 (0.90–1.93)
**Male**					
DDD<140	46	2378.5	1.93	Ref.	Ref.
DDD≥140	43	2489.2	1.73	0.88 (0.58–1.33)	0.93 (0.61–1.42)
**CCI <3**					
DDD<140	12	1222.3	0.98	Ref.	Ref.
DDD≥140	24	1840.7	1.30	1.34 (0.67–2.67)	1.25 (0.62–2.53)
**CCI ≥3**					
DDD<140	81	3836.1	2.11	Ref.	Ref.
DDD≥140	82	3554.2	2.31	1.09 (0.80–1.48)	1.08 (0.79–1.48)

CHR: Crude hazard ratio, The median of cumulative per year dose: 140, HF, Heart Failure

AHR (adjusted hazard ratio): Controlling the variables of age, gender, income, region, urbanization, cci *: p<0.05 **: p<0.001

a: person-years at risk per 100 person-years

DDD, defined daily dose

**Table 5:** Incidence and CHR, AHR for cancer among the sampled patients, according to cancer type, during 8-year followed up period

Presence of cancer	Total(n=4,161)	HF with Digoxin(n=1,219)	HF with Non-Digoxin(n=2,942)
**Lung Cancer (n,%)**	125, 3.00%	42, 3.45%	83, 2.82%
Incidence per 100 person-years	1.54	1.75	1.46
CHR^a^ (95% CI)	−	1.38(1.11–1.7)*	1.00
AHR^b^ (95% CI)	−	1.21(0.98–1.50)	1.00
**Breast Cancer (n,%)**	30, 0.72%	8, 0.66%	22, 0.75%
Incidence per 100 person-years	1.43	1.44	1.43
CHR^a^ (95% CI)	−	1.47(1.18–1.83)**	1.00
AHR^b^ (95% CI)	−	1.30(1.05–1.62)*	1.00
**Liver Cancer (n,%)**	97, 2.33%	34, 2.79%	63, 2.14%
Incidence per 100 person-years	1.49	1.61	1.42
CHR^a^ (95% CI)	−	1.46(1.17–1.83)**	1.00
AHR^b^ (95% CI)	−	1.29(1.03–1.62)*	1.00
**Colorectal Cancer (n,%)**	149, 3.58%	43, 3.53%	106, 3.60%
Incidence per 100 person-years	1.57	1.67	1.54
CHR^a^ (95% CI)	−	1.13(0.80–1.62)	1.00
AHR^b^ (95% CI)	−	1.01(0.70–1.44)	1.00
**Prostate Cancer (n,%)**	59, 1.42%	14, 1.15%	45, 1.53%
Incidence per 100 person-years	1.63	1.55	1.66
CHR^a^ (95% CI)	−	0.85(0.47–1.55)	1.00
AHR^b^ (95% CI)	−	0.75(0.41–1.37)	1.00
**Gynecology Cancer (n,%)**	34, 0.82%	14, 1.15%	20,0.68%
Incidence per 100 person-years	1.85	2.26	1.65
CHR^a^ (95% CI)	−	1.87(0.94–3.70)	1.00
AHR^b^ (95% CI)	−	2.01(1.02–4.20)	1.00
**Bladder Cancer (n,%)**	32, 0.77%	8, 0.66%	24, 0.82%
Incidence per 100 person-years	1.48	1.65	1.44
CHR^a^ (95% CI)	−	0.95(0.42–2.11)	1.00
AHR^b^ (95% CI)	−	0.82(0.37–1.84)	1.00
**Kidney Cancer (n,%)**	30, 0.72%	5, 0.41%	25, 0.85%
Incidence per 100 person-years	1.52	1.87	1.46
CHR^a^ (95% CI)	−	0.55(0.21–1.45)	1.00
AHR^b^ (95% CI)	−	0.47(0.18–1.24)	1.00
**Oral Cancer (n,%)**	18, 0.43%	5, 0.41%	13, 0.44%
Incidence per 100 person-years	1.39	1.33	1.42
CHR^a^ (95% CI)	−	1.13(0.40–3.18)	1.00
AHR^b^ (95% CI)	−	1.12(0.39–3.18)	1.00
**Other Cancer (n,%)**	672, 16.15%	195, 16.00%	477, 16.21%
Incidence per 100 person-years	1.62	1.78	1.56
CHR^a^ (95% CI)	−	1.14(0.96–1.35)	1.00
AHR^b^ (95% CI)	−	1.03(0.87–1.21)	1.00

^a^CHR: Crude hazard ratio was calculated by using stratified Cox proportional hazards regression, HF: Heart Failure

^b^.AHR (adjusted hazard ratio): Controlling the variables of age, gender, income, region, urbanization, cci, *: p<0.05 **: p<0.001

ICD-9-code: Prostate Cancer:185 Liver Cancer:155 Gynecology Cancer:180.182.183 Breast Cancer:174 Lung Cancer:162 Colorectal Cancer:153.154 Oral Cancer:145.141 Bladder Cancer:188 Kidney Cancer:189 Gall bladder Cancer:156

### The proliferative effects by digoxin on the various breast cancer cell lines

**Figure 1:**
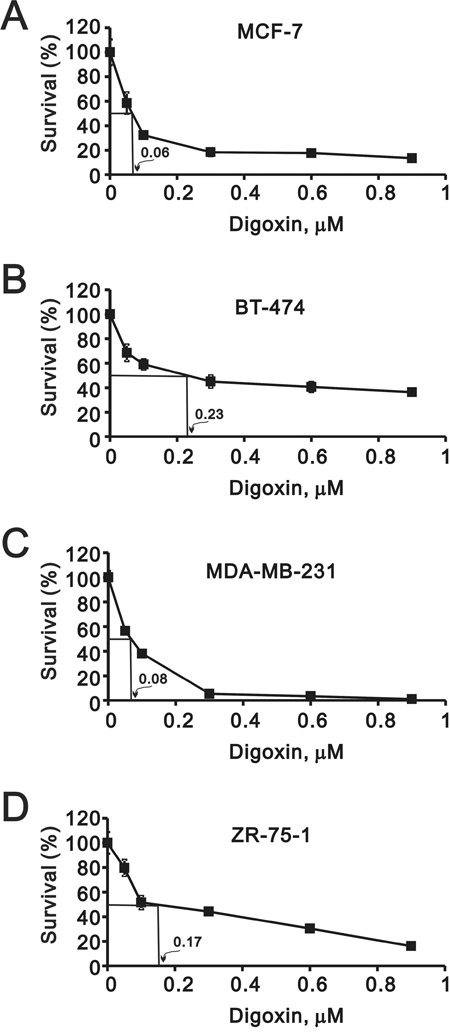
Potential effects of digoxin on the growth rate of various breast cancer cells Breast cells **(A)** MCF-7, **(B)** BT-474, **(C)** MDA-MB-231, and **(D)** ZR-75-1 were treated with indicated concentrations of digoxin for 72 h, MTS assay was performed at the time indicated. The IC50 value was shown in the plot.

Digoxin resembles estrogen chemically and may have an estrogenic effect. It is better to understand whether digoxin has a positive effect on breast cancer cell proliferation. We examined four well-known breast cancer cell lines, including MCF7, BT474, MDA-MB-231, and ZR-75-1. MCF7 and ZR-75-1 cells are categorized into the luminal A subtype; BT474 cell line is categorized into the luminal B subtype; and MDA-MB-231 is categorized into the basal-like subtype. We first analyzed and measured the IC50 value of digoxin in the four breast cancer cell lines. The IC50 value was 60 nM for MCF7 cells; 230 nM for BT474 cells; 80 nM for MDA-MB-231 cells; and 170 nM for ZR-75-1 cells (Figure 1).

**Figure 2:**
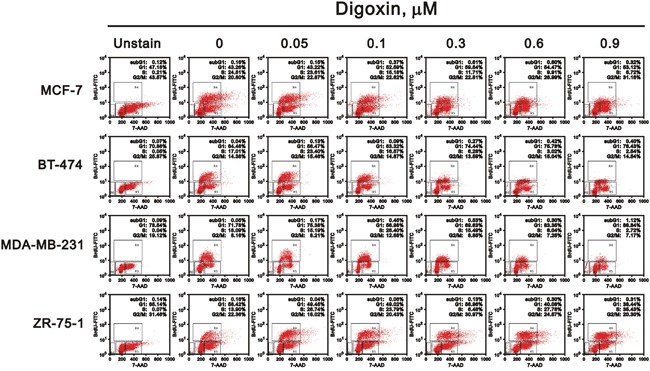
Potential effects of digoxin on the cell cycle profile of various breast cancer cells Four breast cells (MCF-7, BT-474, MDA-MB-231, and ZR-75-1) were treated with indicated concentrations of digoxin for 48 h. The cells were collected and subjected to the 7-AAD staining flow-cytometry analysis to determine the population of various cell cycle phases. The results are representative of two independent experiments.

**Figure 3:**
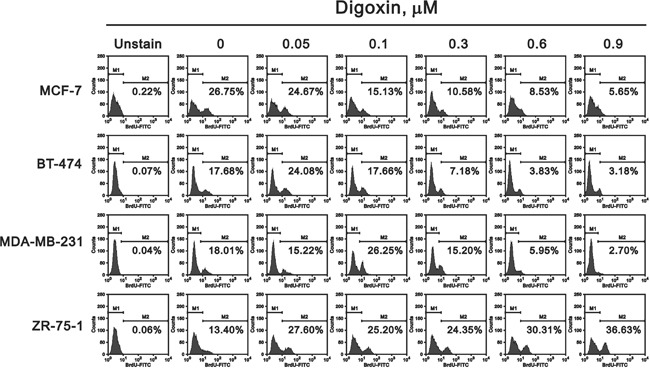
Potential effects of digoxin on the proliferation of various breast cancer cells The same cells shown in Figure 2 were collected and subjected to the BrdU flow-cytometry analysis for the determination of the proliferation rate (M2). The results are representative of two independent experiments.

The cell cycle profile of these four cell lines were treated with digoxin and analyzed via the BrdU-FITC/7-AAD staining method (Figure 2). The ZR-75-1 cell line was the only breast cell line to consistently increase the S phase population by digoxin, accompanied with a decreasing G1 phase population. In contrast, MCF7, BT474, and MDA-MB-231 cells decreased the S phase population by digoxin, accompanied with an increasing G1 phase population. We further verified the digoxin effect on the proliferation of breast cancer cell via the BrdU incorporation from Figure 2 (Figure 3). These findings reconfirmed that digoxin had the ability to induce the proliferation of ZR-75-1 cells, but not in the other three cell lines.

**Figure 4:**
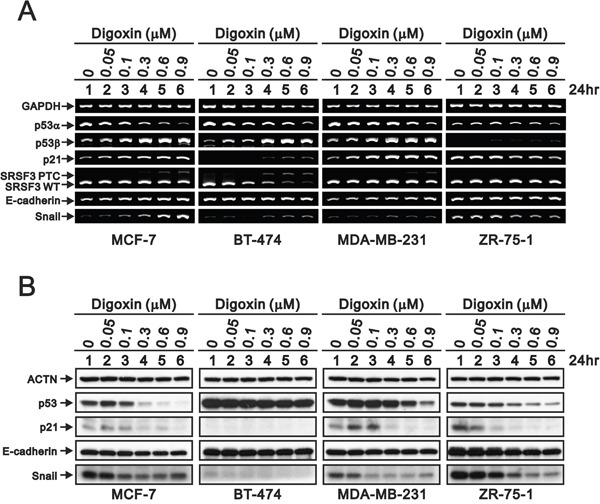
Potential effects of digoxin on the target mRNAs and proteins in breast cancer cells Four breast cells were treated with indicated concentrations of digoxin for 24 h. The cells were collected and subjected to **(A)** RT-PCR analysis of p53α, p53β, p21, SRSF3, E-cadherin, Snail, and GAPDH (loading control) expression, **(B)** the Western blot analysis for the detection of p53, p21, E-cadherin, Snail, and ACTN (loading control). The results are representative of two independent experiments.

Our previous work provided several lines of evidence for digoxin to function in cellular cytotoxicity via the SRSF3-dependent pathway. ZR-75-1 cells were the only tested breast cell line to fit the increasing risk of breast cancer by digoxin. Compared with four breast cancer cell lines using RT-PCR and Western blotting analysis, there was no apparent *SRSF3* premature truncated construct and *p53*β PCR bands in ZR-75-1 cells (Figure 4). In addition to the degradation of p53 and p21 proteins by digoxin, *Snail* was the new target gene and protein in ZR-75-1 cells, as well as MCF7 and MDA-MB-231 cells. In the Western blotting analysis, there was no change of p53, p21, and snail proteins in BT474 cells.

## DISCUSSION

Digoxin has Na^+^/K^+^-ATPase-independent effects [1, 2]. Cumulative studies support the anti-tumor functions of digoxin. Our previous studies have revealed that they deplete SRSF3 protein as well as at the post-transcriptional level via downregulation of SRSF3 gene and protein expression which alternatively splices p53 gene and protein from α into β isoform [8, 20–22]. Up to now, there are many retrospective studies, including epidemiological and clinical, that have elucidated most cases and indicated that the use of digoxin decreased incidences [11, 16–18, 23–26]. In this study, we used clinical information from the NHI program of Taiwan to find several cancers, such as breast, liver, and lung, were positively correlated with the digoxin treatment. The trend is against our previous work, which revealed the anti-tumor function of digoxin in several cancer cell lines, but is consistent with many studies [8, 15, 23, 25]. Furthermore, we verified the potential role of digoxin in four breast cancer cell lines as follows: MCF-7, BT474, MDA-MB-231, and ZR-75-1. Our data reveals that digoxin had the ability to induce the proliferation of ZR-75-1 cells, not the other three cell lines; because there is no SRSE3/p53 pathway. Our findings support the notion that breast cancer encompasses a group of heterogeneous diseases, which can be demonstrated at the molecular, histopathologic, and clinical levels.

The discovery of five distinct subtypes of breast carcinomas (Luminal A, Luminal B, HER2 over-expression, Basal-like, and Normal-like) using whole-genome analysis; each with unique recognizable phenotypes and clinical outcomes [27]. Up to now, 17 commonly used breast cancer cell lines (468, AU 565, BT-20, BT-474, BT-483, HBL101, HS598T, MCF-7, MCF-10A, MCF10F, MCF-12A, MDA-MB-231, MDA-MB-231-UR, MDA-MB-453, MDA-MD-435, SKBR-3, and ZR-75-1) are cultured in an appropriate medium [27, 28]. Based on the expression of ER, progesterone receptor (PR) and HER 2, BT-474 is triple positive, MDA-NA-231 is triple negative, and MCF-7 and ZR-75-1 are positive of ER and PR in this study. The inconsistent proliferation response by digoxin in MCF-7 (luminal A) and ZR-75-1 (luminal B) cell lines was found, suggesting the heterogeneity of gene might play an important role in these two cell lines.

Clinical and biologic heterogeneity in breast carcinomas is also present among the different breast cancer cell lines, and can be revealed by gene expression profiling and IHC analysis [27, 28]. In addition to ER, PR, and HER2, many genes, including p53, have become important predictors and prognostic markers for breast cancer. The work of the Troester group has demonstrated that MCF-7 and ZR-75-1 cells showed a stronger p53-regulated signature following doxorubicin treatment [29]. These inherent differences in p53-dependent targets could lead to different selection pressure for p53 loss in these two wild-type p53 cell line, suggesting that intrinsic heterogeneity of p53 signaling across breast cancer subtypes. In addition to p53 protein itself, Weiss's work has identified that a molecular bypass Snail1 suppresses the anti-proliferative and pro-apoptotic effects exerted by wild-type p53 in breast cancer, revealing that Snail1-dependent regulation of p53 activity probably impacts multiple arms of the carcinoma phenotype [30]. A population-base study showed the divergent rates of p53 mutation by breast cancer subtype and the prevalence of the basal-like and luminal A subtypes was strongly influenced by race and menopausal status [17]. Our work suggests that the SRSF3-p53 pathway might be another possibility to verify this in the future.

The current study had some limitations. Our nationwide population-based data from Taiwan might not be generalizable to other ethnic groups. In addition, this database lacked information of smoking, pregnancies, dietary habits, and other stressful psychosocial events which could be related to cancers. However, our study explored a large sample in Taiwan, and used a reliable model to focus on the relationship between digoxin and cancers.

Our cell-line based results may not be clinically relevant to the general breast cancer population [27]. However, this strategy allows us to consider some possibilities from the clinical findings. The strategy of combining clinical big data with necessary basic research will help us focus on the detailed mechanisms to understand complicated clinical issues and drug usage.

## MATERIALS AND METHODS

### Data source

This study utilized data retrieved from the National Health Insurance Research Database (NHI-RD) between January 1, 2000 and December 31, 2000, which contains all the data regarding claims from Taiwan's NHI program. All medical care providers are required to provide outpatient and inpatient services to those insured to claim their fees from the NHI program [19]. The medical claims information includes data on patient demographics, clinical details (disease and procedure codes according to the International Classification of Disease 9^th^ version (ICD-9-CM)), and healthcare use (days in hospital, drug use, and charges). The National Health Research Institute (NHRI) maintains the database and releases identified secondary data to scientists and clinicians for research purposes.

### Study sample

This retrospective study included a study cohort and a comparison cohort. The study cohort was aged > 18 years with a first diagnosis of heart failure (ICD-9-CM codes 428.xx, 398.91, 402.01, 402.11, 402.91, 404.00, 404.01, 404.03, 404.10, 404.11, 404.13, 404.90, 404.91, 404.93) between January 1, 2000 and December 31, 2000. Our study cohort are patients with heart failure who have taken digoxin (pHF-Digo) pHF-Digo. To ensure the study cohort consists of patients with a diagnosis of heart failure disease and digoxin consumption, patients were included if they had been prescribed DDDs of digoxin in 2000. Also, patients were considered as having continued digoxin therapy if less than 3 months had elapsed between prescriptions [31]. The digoxin included DIGOXIN INJECTION, LANOXIN DIGOXIN TABLETS, LANOXIN DIGOXIN INJECTION, or CARDIACIN ELIXIR [32]. Since the maintenance dose of LANITOP TABLETS is 0.1mg/day, which differs from 0.125~0.75mg/day of the other four types of digoxin [33], we excluded LANITOP TABLETS to avoid bias. The first prescription of digoxin for each patient was assigned as the index date. The exclusion criteria for pHF-Digo were as follows: (1) a previous diagnosis of heart failure before 2000; (2) with a previous cancers index date; and (3) the cancer(s) index date was within 2 years after a prescription for digoxin. The reason or this is that there is likely a latency period of at least 2 years between exposure and the development of a clinically significant cancer [34]. The study cohort included 1,219 patients.

The comparison cohort (pHF) was extracted from the remaining beneficiaries of the database in and included patients aged >18 to limit the study to an adult population. The urbanization level of the residence for each patient was defined as urban, suburban, or rural [35]. Urbanization was evaluated to ensure that the patients had reasonably similar socioeconomic characteristics. Information on preexisting comorbidities was acquired from CCI scores. The CCI is a scoring system that assigns points from 1–6 to a range of diseases [36]. A CCI score of 3 was used as the cutoff score to divide the subjects into two groups according to the sum of their points: < 3 points (low comorbidity) and ≥ 3 points (high comorbidity) [37]. The index date of a (pHF) was defined as the first use of medical care within the index year for each patient. The comparison cohort included 2,942 patients.

### Variables of interest

The risk of cancer (ICD-9-CM codes 140.XX-208.XX) was evaluated for each patient. Patients with heart failure were followed up from their first prescription of digoxin index to the first diagnosis of cancer, death, or the end of follow up. The patients were individually followed up for 10-year medical records.

### Statistical analysis

SAS statistical package (SAS systems for Windows, version 9.2; Cary, NC, USA) was used to perform analyses. Demographic information such as age, sex, income, residential region, and urbanization level was acquired from each patient's file in the NHI database. Age was divided into 3 groups: < 45, 45–65, and > 65 years. Average monthly income was divided into 3 groups: < US$640 (New Taiwan Dollars (NTD) 40,000), US$ 640–1280 (NTD 20,000–39,999), and ≥ US$ 1281 (≥ NTD 40,000). The residential areas of patients were divided into the 4 regions of Taiwan: northern, central, southern, and eastern. The urbanization level was divided into urban, suburban, and rural. An incident case was defined as a patient who was using NHI services for the first time, was diagnosed as a new case with a heart failure disease in 2000, and had not previously been diagnosed with cancer. The incidence density was calculated as the number of incident cases relative to the number per 100 person-years contributed by the subjects of this study. All patients were followed from January 1, 2000 to the date of cancer diagnosis and the data were censored at the date of death or emigration or on December 31, 2010, whichever came first [38]. The Kaplan-Meier method and log-rank test were used to assess the differences in the survival rates and curves, respectively. We studied the development of cancers between pHF-Digo and pHF. A univariate Cox proportional hazard regression was conducted to estimate the CHRs of cancers related to heart failure disease. After controlling for potential confounders of cancers, including age, gender, income, urbanization, and CCI score. A multivariate Cox proportional hazard regression model was applied to calculate the AHRs, which indicated the independent effects of long-term digoxin consumption on the hazard of cancers. A *P* value < .05 was considered statistically significant.

### Ethical approval

This study has been granted an exemption from requiring ethics approval by the Taiwan Medical University-Joint Institutional Review Board (approval number: 201405028).

### Cell culture and survival analysis

MCF-7, BT474, MDA-MB-231, and ZR-75-1 cells were cultured in Dulbecco's modified Eagle's medium supplemented with 10% fetal bovine serum and 1% penicillin-streptomycin (Invitrogen, USA). For cell survival analysis using the MTS (3-(4,5-dimethylthiazol-2-yl)-5-(3-carboxymethoxyphenyl)-2-(4-sulfophenyl)-2H-tetrazolium) assay, cells were seeded in 96-well culture plates and could grow for the indicated periods of time. The MTS assay reagent is composed of MTS and the electron coupling agent phenazine methosulphate (PMS). The 400 μl MTS/PMS solution was added to each well and the plate was incubated for 3 h at 37°C. Transfer 100 μl aliquots of each sample and the absorbance at 490 nm was measured using an ELISA plate reader (Multiskan EX, Thermo, USA). As a control, cells treated with media containing no compounds were set as 100% cell survival.

### Fluorescence-activated cell sorting (FACS) and proliferation analysis

For cell cycle evaluation and cellular proliferation analysis, cells were incorporated in bromodeoxyuridine (BrdU) and flow cytometric analysis, and then processed with the FITC-BrdU Flow Kit per the manufacturer's instructions (BD Biosciences) with specific anti-BrdU fluorescent antibodies. Total DNA was stained with 7-aminoactinomycin D (7-AAD). With this combination, two-color flow cytometric analysis permits the enumeration and characterization of cells that are actively synthesizing DNA (BrdU incorporation as the proliferation rate) in terms of their cell cycle position (i.e., G0/1, S, or G2/M phase defined by 7-AAD staining intensities). The data were analyzed using the CellQuest Pro software package (BD Biosciences) as previously described [39].

### Reverse transcription-polymerase chain reaction (RT-PCR)

Total RNA was isolated using the TRIsure (BIOLINE, UK) reagent followed by the manufacturer's instructions. One microgram of total RNA was subjected to reverse transcription using MMLV reverse transcriptase (Epicentre Biotechnologies, USA) for 60 min at 37°C. The PCR reactions were run on a GeneAmp PCR system 9700 (Applied Biosystems, USA). All PCR primer sequences (Table 6).

**Table 6:** PCR primers used in this study

Gene name	Primer sequence (5’→3’)
***p53α***	Forward: 5’-GATGAAGCTCCCAGAATGCCAGAG-3’ Reverse: 5’-GAGTTCCAAGGCCTCATTCAGCTC-3’
***p53β***	Forward: 5’-ATGGAGGAGCCGCAGTCAGAT-3’ Reverse: 5’-TTTGAAAGCTGGTCTGGTC-3’
***GAPDH***	Forward: 5’-CTTCATTGACCTCAACTAC-3’ Reverse: 5’-GCCATCCACAGTCTTCTG-3’
***SRSF3***	Forward: 5’-ATGCATCGTGATTCCTGTCCATTG-3’ Reverse: 5’-CTATTTCCTTTCATTTGACCTAGATC-3’
***Snail***	Forward: 5’-ATGCCGCGCTCTTTCCTCGTCAGG-3’ Reverse: 5’-TCAGCGGGGACATCCTGAGCAGCC-3’
***E-cadherin***	Forward: 5’-CCTGGGACTCCACCTACAGA-3 Reverse: 5’-GGATGACACAGCGTGAGAGA-3’
***p21***	Forward: 5’-CTGAGCCGCGACTGTGATGCG-3’ Reverse: 5’-GGTCTGCCGCCGTTTTCGACC-3’

### Western blot analysis

Cell lysates were prepared in lysis buffer (100 mM Tris-HCl of pH 8.0, 150 mM NaCl, 0.1% SDS, and 1% Triton X-100) at 4°C. The cell extracts were separated by SDS-PAGE, transferred onto a polyvinylidene difluoride membrane (Millipore, USA) and detected using antibodies against α-actinin (ACTN), p53, p21, snail (Santa Cruz Biotechnology, USA), and E-cadherin (BD, USA).

## Abbreviations

HF: heart failure
ER: estrogen receptor
NHI: National Health Insurance
NHIRD: National Health Insurance Research database
DDDs: defined daily doses
CCI: Charlson Comorbidity Index
NTD: New Taiwan Dollars
CHRs: crude hazard ratios
AHRs: adjusted hazard ratios
PMS: phenazine methosulfate
BrdU: bromodeoxyuridine
7-AAD: 7-aminoactinomycin D
PR: progesterone receptor
